# Divergence of the mRNA targets for the Ssb proteins of bacteriophages T4 and RB69

**DOI:** 10.1186/1743-422X-1-4

**Published:** 2004-09-17

**Authors:** Jamilah M Borjac-Natour, Vasiliy M Petrov, Jim D Karam

**Affiliations:** 1 Department of Biochemistry SL 43, Tulane University Health Sciences Center, 1430 Tulane Avenue, New Orleans, LA 70112, USA; 2 Lebanese American University, PO Box 13-5053, Mailbox S-37, Beirut, Lebanon

**Keywords:** Ssb protein, gp32, RNA-binding proteins, DNA-binding proteins, translational control, DNA replication

## Abstract

The single-strand binding (Ssb) protein of phage T4 (T4 gp32, product of gene 32) is a mRNA-specific autogenous translational repressor, in addition to being a sequence-independent ssDNA-binding protein that participates in phage DNA replication, repair and recombination. It is not clear how this physiologically essential protein distinguishes between specific RNA and nonspecific nucleic acid targets. Here, we present phylogenetic evidence suggesting that ssDNA and specific RNA bind the same gp32 domain and that plasticity of this domain underlies its ability to configure certain RNA structures for specific binding. We have cloned and characterized gene 32 of phage RB69, a relative of T4 We observed that RB69 gp32 and T4 gp32 have nearly identical ssDNA binding domains, but diverge in their C-terminal domains. In T4 gp32, it is known that the C-terminal domain interacts with the ssDNA-binding domain and with other phage-induced proteins. In translation assays, we show that RB69 gp32 is, like T4 gp32, an autogenous translational repressor. We also show that the natural mRNA targets (translational operators) for the 2 proteins are diverged in sequence from each other and yet can be repressed by either gp32. Results of chemical and RNase sensitivity assays indicate that the gp32 mRNA targets from the 2 related phages have similar structures, but differ in their patterns of contact with the 2 repressors. These and other observations suggest that a range of gp32-RNA binding specificities may evolve in nature due to plasticity of the protein-nucleic acid interaction and its response to modulation by the C-terminal domain of this translational repressor.

## Introduction

T4 gp32, the single-strand binding (Ssb) protein of bacteriophage T4, is a well studied member of the Ssb protein family, and was the first such ssDNA-binding replication protein to be discovered [1]. The protein, product of T4 gene 32, is an essential component of the phage DNA replication complex and also plays essential roles in DNA repair and recombination [2,3]. Like other Ssb proteins, T4 gp32 facilitates transactions at the replication fork, especially along the lagging strand, through its binding to the unwound DNA template and its specific interactions with other protein components of the DNA replisome. T4 gp32 is known to stimulate the phage induced DNA polymerase (T4 gp43) and to play a role in the dynamics of primosome (T4 gp61-gp41 complex) recruitment by the primase-helicase assembly protein T4 gp59 [4-6]. In general, Ssb proteins lack specificity to the ssDNA sequence and this property allows them to perform their physiological roles at all genomic locations undergoing replication, repair or recombination. The presence of a Ssb protein in the right place at the right time may depend, in large measure, on specificity of its interactions with other proteins from the same biological source.

T4 gp32 has the interesting property of being able to control its own biosynthesis at the translational level *in vivo*. The protein binds to a specific target (translational operator) in the 5' leader segment of the mRNA from gene 32, and represses translation of this RNA [7]. Another Ssb protein, gp5 of the M13 ssDNA phage family, has also been shown to act as a mRNA-specific translational repressor, although in this case, the RNA target is located in the message for another essential M13 replication protein, gp2 (an endonuclease) [8,9]. It is not known if other Ssb proteins, especially those for cellular DNA replication and maintenance, also possess RNA binding functions that regulate specific translation or other physiologically important RNA-dependent processes. In T4, the physiological link between the sequence-independent ssDNA and specific RNA binding functions of gp32 has been explained by a model based on *in vitro *measurements of the protein's binding affinities to different nucleic acid ligands. It has been observed that ssDNA is favored over translational operator RNA as a ligand for T4 gp32 and that RNA of nonspecific sequence is the least preferred nucleic-acid ligand for this Ssb protein [10-12]. *In vivo*, T4 encoded mRNA for gp32 is intrinsically more metabolically stable than the typical prokaryotic mRNA and is thought to have opportunities to undergo many cycles of gp32-mediated repression and depression during the replication and other processing of phage DNA. The potential for translation of this mRNA in the T4 infected *E coli *host is thought to be determined by availability of ssDNA in the metabolic pool [10,13,14]. DNA damage or unwinding transactions are thought to draw gp32 away from its mRNA target to the exposed ssDNA, thus causing derepression of translation and upward adjustments in gp32. Repression of the mRNA would then be reestablished if the amount of gp32 exceeded the number of exposed ssDNA sites for the protein. This model is consistent with many *in vivo *observations relating to levels of T4 gp32 biosynthesis under conditions of DNA damage or abnormal accumulation of ssDNA in the phage infected bacterial host [7].

It is not clear how T4 gp32 distinguishes between specific RNA and the non-specific nucleic acid sequence of ssDNA or ssRNA ligands. It appears that single-strandedness of the nucleic acid is not the most important criterion used by the protein to selectively bind its own message in the phage-induced mRNA pool. The translational operator for T4 gp32 has been mapped by RNA footprinting assays and determined to consist of two contiguous components, a 5' terminal ~28-nucleotide component that forms a folded structure (RNA pseudoknot) and an adjacent, less structured, >40-nucleotide component that lies 3' to the pseudoknot [15,16]. The 3' terminal component includes several repeats of UUAAA or UAAA sequences, in addition to harboring typical prokaryotic nucleotide determinants for translation initiation by ribosomes [7,16,17]. The RNA pseudoknot and UUAAA/UAAA elements are both essential for autogenous repression of the mRNA by T4 gp32 [15,16,18]. *In vitro *studies suggest that the pseudoknot serves as the initial recognition (nucleation) site for the protein and that this gp32-RNA interaction leads to cooperative binding of additional gp32 monomers to the less structured downstream sequence containing the UUAAA/UAAA elements and ribosome-binding site (RBS) [16]. Cooperative binding to the mRNA is envisaged to be analogous to gp32-ssDNA interactions, except that the UUAAA/UAAA sequence elements probably contribute to specificity of the mRNA interaction to the protein.

The 3-dimensional structure of intact T4 gp32 has not been solved, although a number of biochemical and physiological observations have provided clues that the protein is modularly organized into 3 distinct domains [19]. In particular, studies with proteolytic fragments of purified T4 gp32, including the analysis of a crystal structure for one of these fragments [20], have assigned the ssDNA binding function to a module formed by an internal segment of the 301-residue protein. It is presumed that this domain is responsible for binding specific RNA as well, although no direct evidence exists for this notion. In the studies described here, we show that the ssDNA-binding domain is highly conserved between T4 gp32 and the phylogenetic variant of this protein from the T4-like phage RB69. Yet, we also show that sequences of the mRNA targets for the two Ssb proteins are different and that the two repressors differ in their patterns of interaction with these targets. We present results suggesting that specificity of gp32 to RNA has co-evolved with specificity of this Ssb protein to other phage induced proteins of DNA metabolism that interact with gp32's C-terminal domain. Our studies suggest that the ability of a diverging regulatory RNA to make alternate contacts with a mutually plastic, but highly conserved, RNA-binding protein site may allow the RNA to tolerate mutational changes without loss of the regulatory function. Such plasticity of the interacting partners could allow for the evolution of a broad spectrum of gp32-RNA binding specificities despite selective pressures that conserve the amino acid sequence of the protein's nucleic acid-binding domain.

## Methods

### Bacterial and phage strains used

The *E coli *K-12 strain K802 (*hsdR*, *hsdM*^+^, *gal*, *met*, *supE*) was used as host in cloning experiments and the *E coli *B strain NapIV (*hsdR*_*k*_^+^, *hsdM*_*k*_^+^, *hsdS*_*k*_^+^, *thi*, *sup*^*o*^) was the host for plasmid-mediated gene expression studies that utilized lambda *pL *control. *E coli *B strain BL21(DE3), which harbors a T7 RNA polymerase gene under cellular *lac *promoter control [21], was used as the host for T7 Φ10-promoter plasmids in pilot experiments that assessed toxicity of cloned RB69 gene *32 *to bacterial cells.

### Cloning and nucleotide sequence determination of RB69 gene *32*

**Figure 1:**
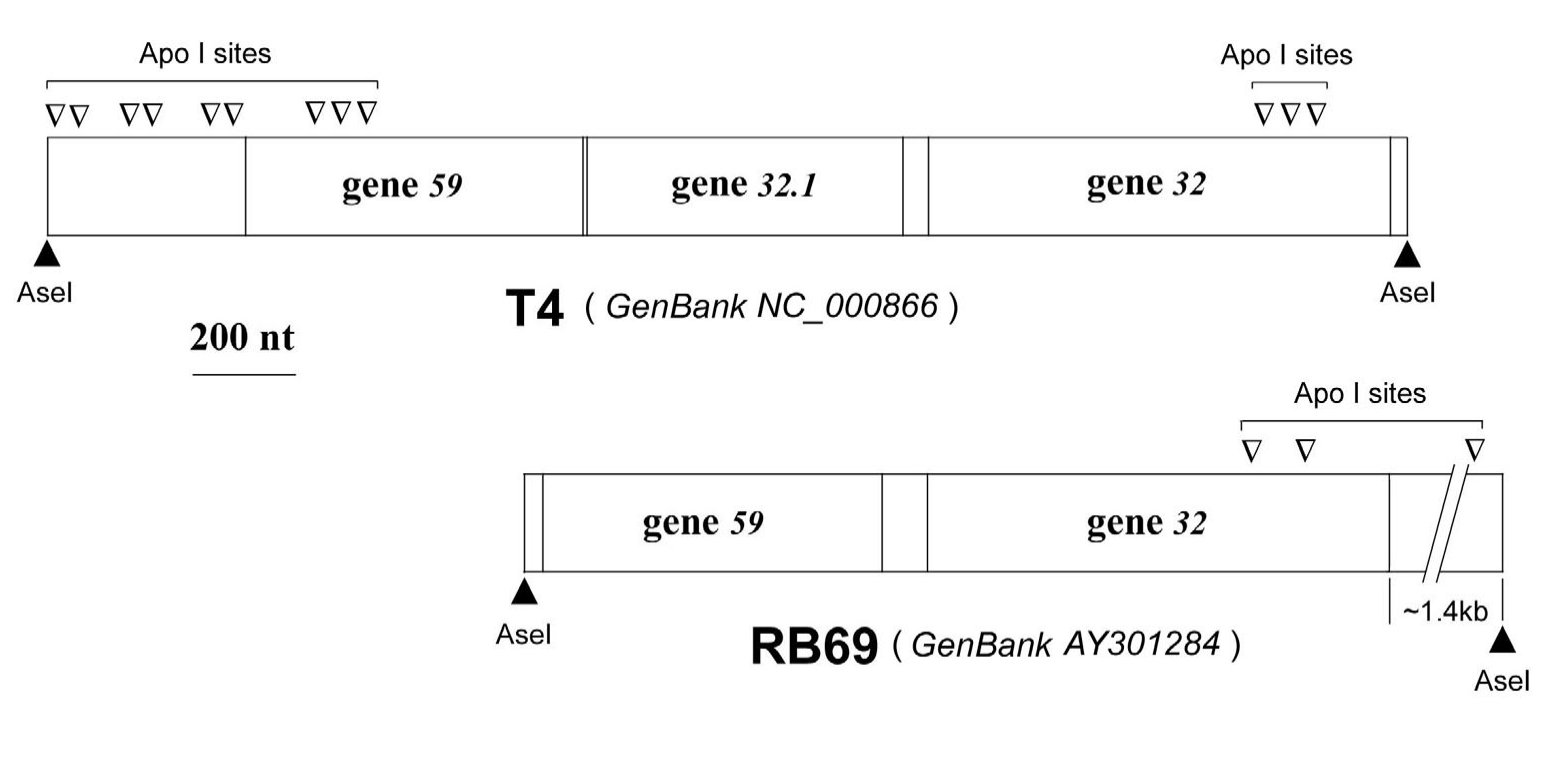
**A comparison between the genetic maps of the Ssb protein (gp32) encoding regions of phages T4 and RB69. **Note the presence of an open-reading frame (ORF) for a homing endonuclease (SegG protein; [45]) between T4 genes 59 (gp59; primase-helicase loader) and 32 (gp32; Ssb protein). The restriction sites we used for cloning RB69 gene 32 are marked, and compared to the locations of analogous sites in T4. GenBank Accession numbers for the genetic regions of interest are also noted.

In preliminary experiments, we used Southern blot analysis of *AseI*-digested RB69 genomic DNA to identify and retrieve an ~35-kb DNA fragment that hybridized to a T4 gene *32*-specific riboprobe under stringent conditions. The riboprobe was prepared by methods described previously [22,23] using the T4 gene *32 *clone pYS69 [15], which was generously provided by Y Shamoo. We were unable to clone this *AseI *fragment in *AseI*-compatible *Eco *R1-generated ends of plasmid vectors. However, further digestion of the *AseI *fragment with *ApoI *(which generates *Nde1*-compatible ends) yielded a shorter, ~15-kb, fragment that could be cloned in the *NdeI-EcoRI *interval of vector pNEB193 (cat# N3051S, New England Biolabs, Beverly, MA). The cloned fragment was sequenced and found to be very similar to the T4 genetic segment extending from gene *59 *through the 5' terminal ~2/3 of gene *32*, except that the RB69-derived DNA appeared to lack a homologue of the T4 *ORF 32.1 *(see below). Comparisons between the T4 and RB69 gene *59-32 *regions are diagrammed in Fig 1. We retrieved the remainder (3' terminal segment) of RB69 gene *32 *from RB69 genomic DNA, through PCR amplification using Taq DNA polymerase. For this purpose, we utilized two primers, one perfectly matching a sequence in the cloned *AseI-ApoI *RB69 fragment (ie, upstream primer: 5'GCTGCTAAGAAATTGTTCATAG3') and the other (the downstream primer), an 18-mer bearing the sequence 5'CAGCAGCAGTGAAACCTTTA3', was chosen from a PCR screen of an RB69 primer library. DNA amplification was carried out under low-stringency conditions for primer annealing (30 sec at 25°C), which allowed activity from the imperfectly matched downstream primer. We obtained several products that we resolved by agarose gel electrophoresis Only one of these products, an ~35-kb DNA fragment, hybridized, although poorly, to the T4 gene *32*-specific riboprobe initially used for the Southern blot analysis of *Ase1*-digested RB69 genomic DNA. This fragment was sequenced, using the PCR, and found to contain the 3' terminal segment of RB69 gene *32 *as well as some of the region distal to RB69 gene *32 *(relative to the T4 genetic map). Collectively, sequence analysis of the cloned and amplified RB69 genomic segments yielded sufficient information for designing new primers to amplify, from genomic DNA, the entire wild-type RB69 gene *32*, as well as shorter segments of this gene and its putative control region in the untranslated RB69 *IC59-32 *region (Fig 1). DNA sequence information obtained from these analyses was also used for another study, which was aimed at determining the sequence of the entire RB69 genome (GenBank NC_004928).

### Assays for plasmid directed gene *32* expression

**Figure 4:**
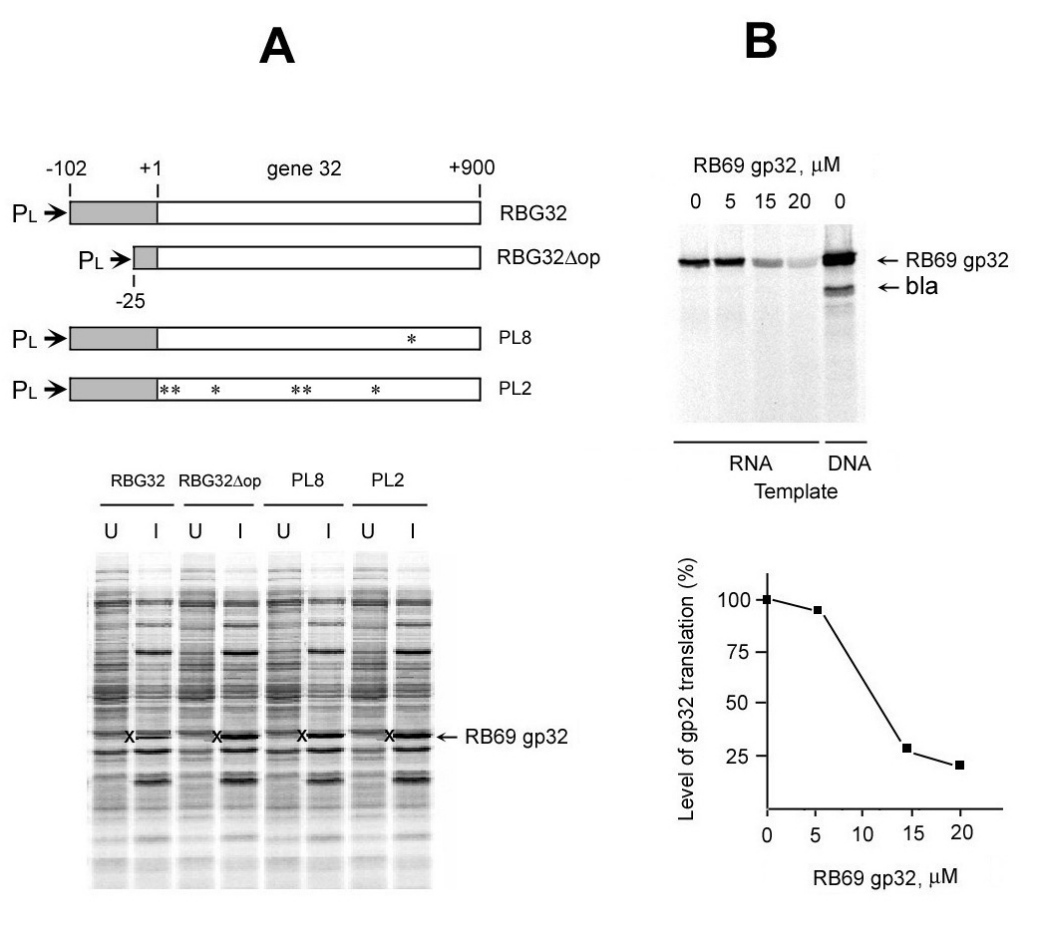
**Results of experiments showing that RB69 gp32 is an autogenous translational repressor. **For Panel A, λ*CI857PLN*-bearing plasmid clones of the diagrammed DNA segments were heat-induced (42°C) and assayed for gp32 synthesis as described in other work [24,27]. RBG32 is a DNA segment that carries the wild-type sequence from -120 through +900 relative to the first base of the initiator AUG of RB69 gene 32. RBG32Δop is a truncated derivative of RBG32 that lacks elements of the putative RNA pseudoknot of RB69 gene 32 (Figs 3 & 6). PL8 is identical to RBG32 except that it carries a single-base substitution (marked with an asterisk) in codon 173, leading to a F173S substitution in RB69 gp32. PL2 is similar to RBG32 and PL8, except that it carries several point mutations (map positions marked with asterisks). Panel B shows results of an experiment in which purified RB69 gp32 was shown to inhibit in vitro translation of purified mRNA from the cloned RBG32 fragment, as well as mRNA from in vitro expressed plasmid clone (coupled transcription/translation). Conditions for these assays are described in METHODS.

We used the lambda *pL *plasmid vector pLY965 [24] to clone RB69 gene *32 *sequences that were designated for *in vivo *expression studies. This vector expresses cloned DNA under control of the heat-inducible λ*cI857pL *element, which produces sufficient *cI857 *repressor under uninduced conditions (≤30°C) as to maintain *pL*-mediated expression at undetectable levels. Minimizing plasmid-driven transcription from *pL *contributed to stable maintenance of the cloned wild-type RB69 gene *32*, the product of which is highly toxic to bacterial cells. RB69 gene *32 *mutants still emerged when such clones were grown at ≤30°C. Some of these mutants were archived for use as controls in certain studies (eg, PL2 and PL8, Fig 4). With the T7 Φ10-promoter expression vector pSP72 (Promega) as the cloning vehicle, clones containing the wild-type RB69 gene *32 *were not viable when introduced into *E coli *BL21(DE3), probably because of residual (constitutive) *lac*-promoter activity in this bacterial host. To circumvent potential toxicity, pSP72-based recombinants were propagated in hosts lacking a T7 RNA polymerase gene. The purified plasmid DNA from these hosts was used for *in vitro *transcription and translation assays. Methods for the radiolabeling of plasmid encoded proteins and their subsequent analysis by SDS-PAGE have been described elsewhere [24,25], and conditions pertaining to specific experiments are given in figure legends.

### Purification of gp32 from clones of the structural gene

RB69 gp32 and T4 gp32 were purified from the overproducing clones pRBg32Δop (RB69 gp32) and pYS69 (T4 gp32), respectively. We used the gp32 purification protocol outlined by Bittner et al, [26] with minor modifications. The preparation of crude extracts, from 6-liter batches of heat-inducible *E coli *NapIV clones of phage genes, was as described previously for T4 RegA protein [27]. Anionic-exchange chromatography (using Q-Sepharose; Cat# 17-0510-01; Pharmacia) was as described for purification of plasmid-generated RB69 gp43 [28]. Under the conditions used, gp32 eluted at 0.3–0.4 M NaCl. In the subsequent chromatographic step, utilizing Phenyl-Sepharose (Cat#17-0965-05; Pharmacia), we tested column fractions for nuclease contamination by incubating 4 μl samples with plasmid DNA (~1 μg) overnight at room temperature and then analyzing the mixtures by agarose gel electrophoresis. The gp32-containing fractions that exhibited no hydrolysis of the plasmid DNA were pooled and the protein was purified further by chromatography on ssDNA-agarose (Cat #15906-019; Invitrogen). Pooled fractions from the ssDNA chromatography were dialyzed against a gp32 storage buffer containing 0.1 M NaCl, 20 mM Tris-HCl pH 8.0, 1 mM EDTA, 0.5 mM DTT and 50% glycerol Protein stocks (at 4–8 mg gp32/ml) were stored at -20°C until used.

### Preparation of RNA for in vitro studies

RNA preparations used for footprinting and other *in vitro *studies originated from *in vitro *transcription of pSP72 clones of the desired gene 32 sequences Methods have been described elsewhere [29]. Phage-specific RNA sequences of the purified transcription products used for footprinting included nucleotide positions -102 to +161 (relative to the initiator AUG) in case of the RB69 gene *32 *transcripts and positions -96 to +161 in case of the T4 gene 32 transcripts. These products also included a 10-nt sequence from the plasmid's T7 promoter region RNA sequencing was carried out by using the RVT-catalyzed primer-extension (cDNA synthesis) method described elsewhere [23,29]. Sequencing primers were annealed to codons 12 to 20 of the transcripts and the sequenced segments of the RNA spanned nucleotide positions +36 through about -100 relative to the initiator AUG. For *in vitro *translation assays, the RNA preparations included full length and truncated versions of the gene *32 *open-reading frame from each of the 2 phage sources.

### Assays for gp32-mediated in vitro translational repression

We used E coli S30 cell-free extracts (Cat#L1020; Promega) with purified pSP72-based gene *32 *recombinant DNA (coupled transcription-translation assays) or purified RNA (DNA-free translation assays) to assess repressor activities of purified RB69 gp32 and T4 gp32. With plasmid-directed gene *32 *expression, it was possible to use expression of the plasmid borne *bla *gene (β-lactamase) as an internal control. Each 50 μl *in vitro *assay reaction mixture (placed in a 15-ml conical tube) contained 1 μg of plasmid DNA template or 4 μg RNA, 5 μl of a mixture of all amino acids (1 mM each) except L-methionine, 1 μl of an S30-premix cocktail (containing rNTPs, tRNAs, an ATP generating system and required salts), 15 μl S30 extract and the balance of volume in nuclease-free water Reaction mixtures, including any added gp32, were constituted in an ice bath before transferring to 37°C for incubations (30 or 60 min). Reactions were stopped by rechilling in the ice bath. Proteins from 5 μl samples were precipitated with 20 μl acetone, collected by centrifugation, dried and suspended in SDS extraction buffer for analysis by SDS-PAGE and autoradiography. Analysis of plasmid encoded (N-terminal) gp32 fragments was carried out in SDS-PAGE (10% gels) using Tricine as the electrophoresis buffer. This buffer system allows for effective resolution of small polypeptides [30]. When used, purified gp32 was added at concentrations ranging between 5 and 20 μM.

### Treatments of RNA with RNases and chemical agents

The RNA-modifying chemical reagents Dimethylsulfate (DMS; Cat# D18,630-9; Aldrich) and Diethylpyrocarbonate (DEPC; Cat# D5758; Sigma) and the ribonucleases (RNases A1, T1 and V1 respectively) were used to probe RB69- and T4-derived operator RNAs for intrinsically structured regions. The RNases were also used for RNA footprinting (protection by gp32) studies.

DMS was diluted in absolute ethanol at ratios of 1:2, 1:4, and 1:5 ratio v/v and its effects were analyzed at the three concentrations. The reaction buffer contained 30 mM HEPES pH 7.5, 10 mM MgCl2. Reactions were stopped in 0.5 M β-mercaptoethanol and 0.75 M sodium acetate. The protocol for DEPC treatment was identical to that for DMS, except that we used 1 μl of DEPC per 100 μl of reaction mix and incubated the reactions at room temperature for 10 min.

For the RNase-sensitivity assays, including gp32-mediated RNA footprinting, digestions with RNases A1 and T1 were carried out in 30 μl buffer containing 60 mM NH_4_Cl, 10 mM Mg acetate, 10 mM Tris-HCl pH 7.4, and 6 mM β-Mercaptoethanol. The buffer for digestions with RNase V1 contained 25 mM Tris-HCl pH 7.2, 10 mM MgCl_2_, and 0.2 M NaCl Incubations were at 37°C in 30 μl buffer in all cases. RNase treatments were halted with an equal volume of buffer containing 0.4 M Na acetate pH 5.2, 20 mM EDTA, and 30 μg *E coli *tRNA. When used for RNA footprinting, RB69 gp32 or T4 gp32 was added at concentrations in the range between 1 μM and 5 μM.

## Results

### A sequence comparison between T4 gp32 and RB69 gp32

**Figure 2:**
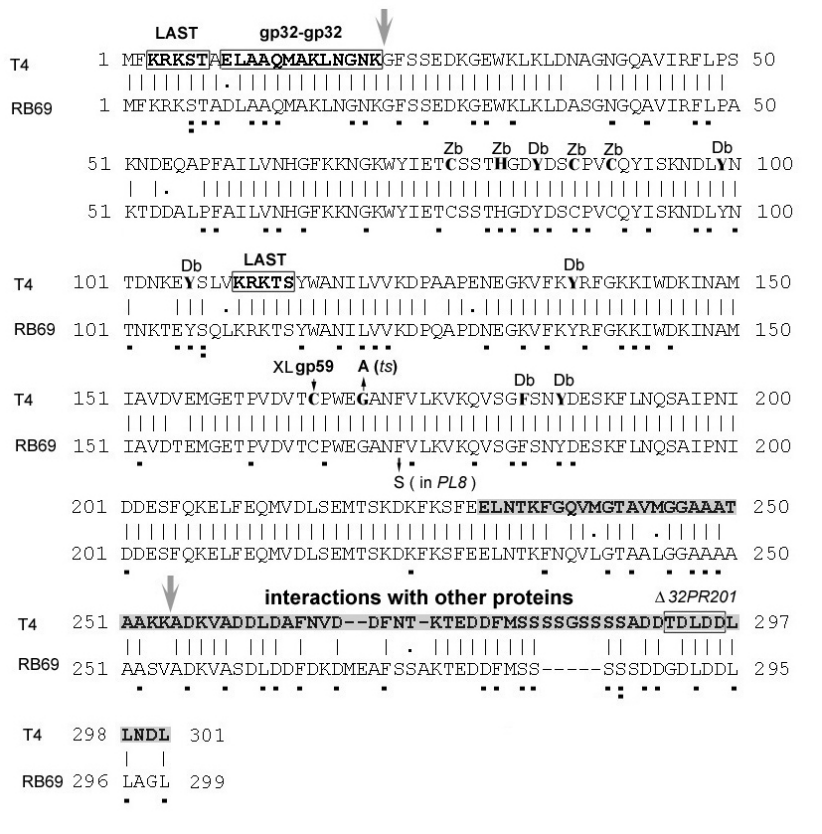
**Amino-acid sequence alignments between the Ssb proteins (gp32s) of T4 and RB69. **Residues and segments of the T4 gp32 sequence that have been implicated in specific biological functions of the protein are marked as follows: Db [DNA binding residue]; Zb (residues that coordinate Zn^++ ^in the zinc-binding domain; [20,46]); gp32-gp32 [residues involved in cooperative gp32 binding to ssDNA]; XLgp59 (residue that cross-links to gp59; [42]); LAST (sequence motifs, (Lys/Arg)3 (Ser/Thr)2, that have been proposed to directly bind nucleic-acids or mediate gp32-gp32 interactions [31]). The shaded C-terminal portion of T4 gp32 has been implicated in interactions with other phage induced proteins [38]. The small deletion (Δ*32PR201*) alters specificity of T4 gp32 in phage replication without affecting autogenous translational repression [39]. The largest vertical arrows denote trypsin-hypersensitive sites (19) The G-to-A mutation marked "(ts)" was isolated in this laboratory as a missense (temperature-sensitive) suppressor of a defective gp43 function (unpublished). In the RB69 gp32 sequence, residues whose codons differ from their conserved T4 counterpart at the third nucleotide are underscored with a single dot; those differing by 2 nucleotides are marked by 2 dots.

The amino acid sequence of RB69 gp32 was deduced from the determined nucleotide sequence of the gene. An alignment between the predicted primary structures of this protein and its T4 homologue is shown in Fig 2, which also highlights the main differences between the 2 proteins and points out certain functionally important landmarks on the T4 gp32 sequence. The two proteins are identical at ~85% of amino-acid positions (92% overall similarity), with most of the differences being clustered in 2 short blocks of amino-acid sequence in the highly charged C-terminal segment of the protein, D264(RB69)/A264(T4) to L299(RB69)/L301(T4). Both C-terminal segments are rich in serines and aspartates; however, they differ in their arrangements of these residues and the serine-rich cluster is 5 residues longer in T4 gp32 (S282-S286). In contrast to their conspicuous differences in the C-terminal domain, T4 gp32 and RB69 gp32 are closely similar in segments that, in T4 gp32, have been implicated in cooperative gp32-gp32 interactions (95% identity/100% similarity for the N-terminal 21 residues) and ssDNA binding (residues 21 to 254; ~92% identity/~95% similarity). We note that all T4 gp32 residues that have been implicated in ssDNA binding are conserved in RB69 gp32 (Fig 2). However, interestingly, codon sequences for the two aligned N-terminal gp32 segments differ at many third nucleotide positions between T4 and RB69, suggesting that there has been natural selection for amino acid identity (and not merely chemical or side-chain similarity) in the N-terminal two-thirds of the phage Ssb protein. We also note that both proteins contain 2 "LAST" (3KRKST7 or 110KRKTS114) sequence motifs, which in the T4 system have been implicated in interactions with the negatively charged surfaces of DNA as well as with the C-terminal domain of gp32 [31]. One of these motifs (K3-T7) lies near the extreme N-terminus of the protein and the second (K110-S114) is adjacent to a short sequence (residues 102–108) that diverges between T4 and RB69 (~50% similarity), but that also contains 3 conserved charged residues including the DNA-binding tyrosine Y106 of T4 gp32 [20].

### The RB69 *IC59-32* region

**Figure 3:**
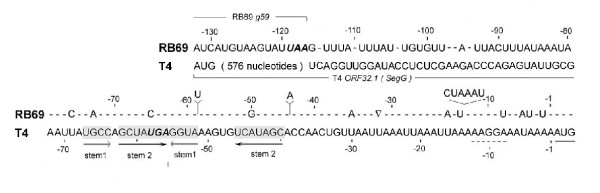
**A comparison between the nucleotide sequences of the T4 *IC32.1*-*32 *and RB69 *IC59*-*32 *regions. **These 2 regions contain determinants for translation initiation of the respective phage-induced mRNAs for gp32. The chart emphasizes sequence differences (entered as lettered residues in the RB69 sequence) between the 2 regions. The dashes indicate identity between RB69 and T4 residues. Sequence elements contributing to RNA pseudoknot formation in the T4 gene 32-specific mRNA are marked by horizontal arrows. Note the sequence overlap between elements of the pseudoknot and *ORF32.1 (segG) *of the T4 sequence. Also, see Fig 6 for a summary of properties of the RB69 sequence.

Figure 3 shows an alignment of the RB69 *IC59-32 *region with its counterpart (the *IC32.1-32 *region) from T4 The T4 region (GenBank NC_00866) has been experimentally documented to harbor the translational operator for gene 32 expression [6]. The RB69 counterpart (GenBank NC_004928) is 7 nucleotides longer and ~70% identical in sequence. By comparison, the gp32 encoding portions of the T4 and RB69 genes are ~80% identical in the overall nucleotide sequence (see Fig 1 for GenBank accession numbers) and their predicted protein products are >90% similar in amino acid sequence. There is an additional 40-nt untranslated sequence in the RB69 *IC59-32 *region that appears to have no T4 counterpart (Fig 3), and *ORF321 *is missing altogether in RB69 (Fig 1). So, it appears that the regions between genes 59 and 32 of T4 and RB69 have undergone more evolutionary divergence from each other than their gp32-encoding regions. However, despite their differences in nucleotide sequence, the translational operator sequence of T4 gene 32 and its putative RB69 counterpart are predicted, by computer programs, to form similar structures. We address this prediction below and present experimental evidence for the RNA structure and its role in translational control of RB69 gp32 synthesis.

### RB69 gp32 and T4 gp32 are functionally similar

Figure 4 shows results from experiments that measured the effects of RB69 gp32 on its own synthesis *in vivo *(Fig 4A) and *in vitro *(Fig 4B). The *in vivo *experiments measured plasmid-directed RB69 gene *32 *expression by *E coli *clones carrying wild-type and mutant versions of the RB69 gene. As shown in Fig 4A, induced expression of the gene was lower (by ~4-fold) with the wild-type construct than with deletion mutants of the untranslated 5' leader of the mRNA (RBG32Δop, Fig 4A) or missense mutants in the structural gene from this phage (PL2 and PL8 constructs; Fig 4A). These observations are consistent with the explanation that RB69 gp32, like T4 gp32, is able to bind and repress its own mRNA. The results shown in Fig 4B confirm that purified RB69 gp32 is a potent repressor of translation of purified mRNA for this protein.

We have used similar experiments to those for Fig 4 to compare repressor activities of T4 gp32 and RB69 gp32 on identical RNA targets, and observed that either protein can repress gene *32*-specific mRNA from either source (results not shown). However, such experiments, which require 10–30 μM purified protein to demonstrate repression (Fig 4B), did not unambiguously distinguish between the RNA-binding specificities of the 2 proteins. Also, in phage-plasmid complementation assays, we observed that the cloned RB69 wild-type gene 32 supported efficient growth of T4 gene *32 *mutants (bursts of ~100) By these criteria, the T4 and RB69 proteins appeared to be similarly functional in each other's physiological systems. Yet, the natural targets for the 2 proteins are clearly different from each other in topography (Fig 3) and as we describe later, RNA-binding specificity differences between the 2 proteins could be detected through *in vitro *RNA-footprinting assays, which utilized lower concentrations of gp32 than is usually required to detect gp32-mediated repression by *in vitro *translational assays.

### RNA structure in the RB69 gene 32 translational initiation region (TIR)

**Figure 5:**
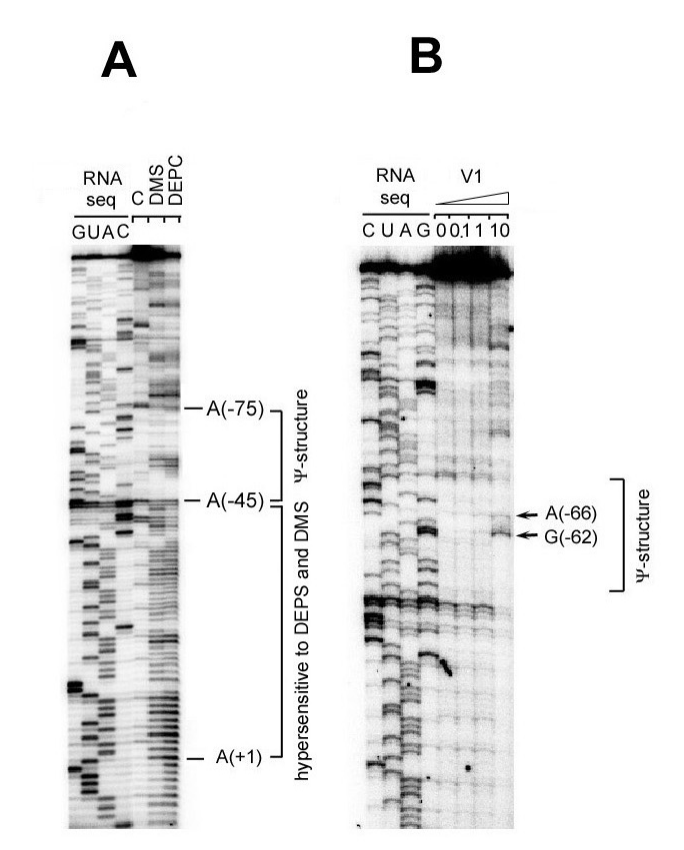
**Portions of autoradiograms from RNA sequencing gels showing sites of cleavage in RB69 gene 32-derived RNA following treatments with DMS and DEPC (Panel A) and RNase V1 (Panel B). **These experiments probed the RB69 RNA for secondary and higher-order structure. The lanes marked "RNA seq" show results from sequencing untreated RNA by the RVT-catalyzed chain termination method [23,35]. In Panel A the lane marked with a "minus" sign shows the positions of RVT chain termination caused by RNA structure in the untreated RNA. The DMS and DEPC lanes show sites of hypersensitivity (cleavage) of the same RNA to treatment with these chemical agents. In Panel B, the V1 lanes denote the amount of RNase V1 (×10^-5 ^units) used to digest the RNA substrate.

**Figure 6:**
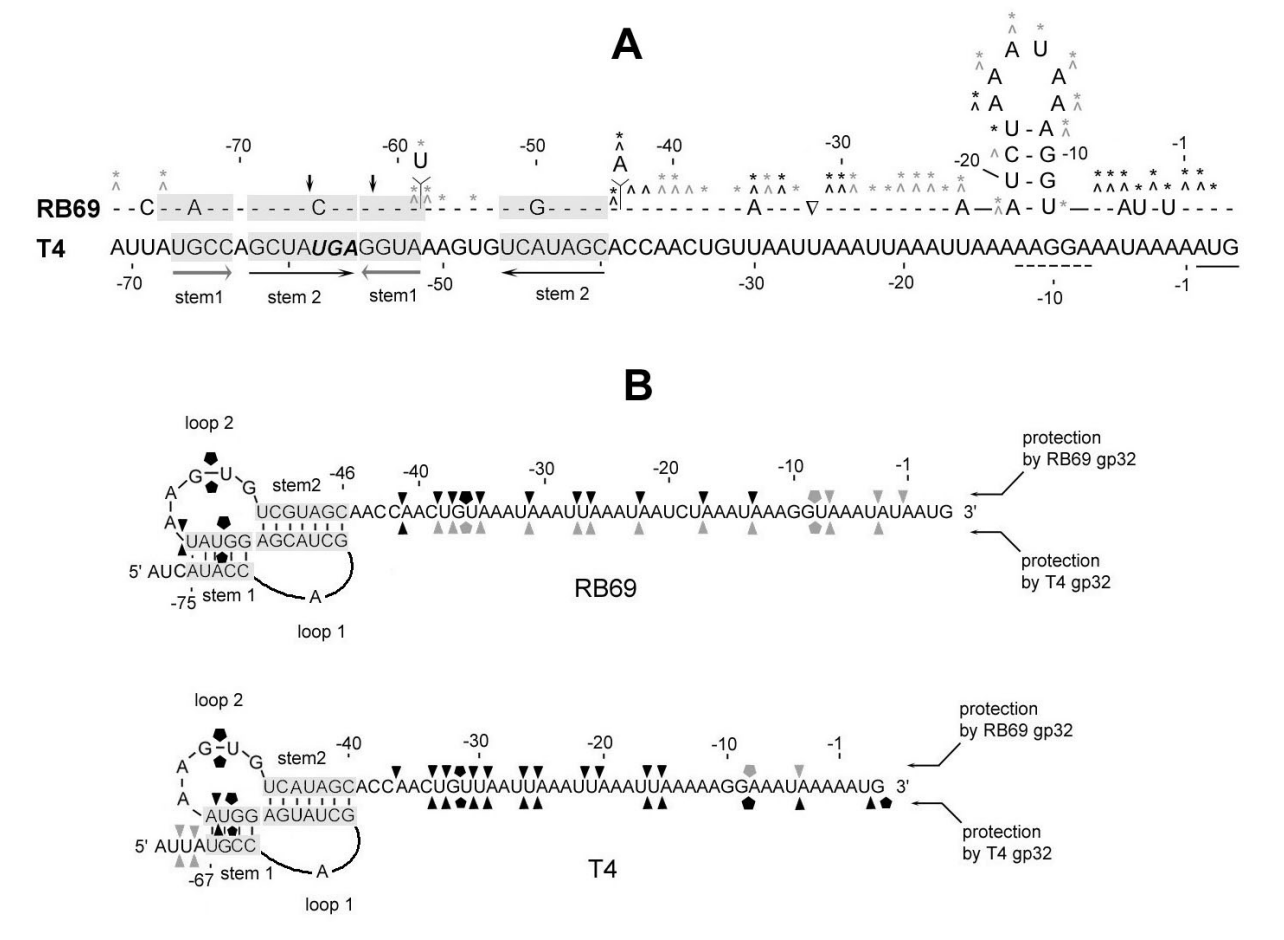
**Summaries of results from the chemical and RNase sensitivity and RNA footprinting studies reported here. **Panel A shows our interpretation of experiments that probed the existence of RNA structure in RB69 gene 32-specific RNA (Fig 5). The T4-derived RNA counterpart is shown for comparison The "caret" symbol denotes sensitivity to cleavage after DMS treatment; asterisks denote sensitivity to cleavage after DEPC treatment. The darker symbols denote greater sensitivity. Positions that are not marked by any symbols were resistant to the modifying agents under the conditions used. Vertical arrows mark positions that were sensitive to RNase V1. Panel B shows our interpretation of the RNA footprinting studies described in Figs 7 and 8. Positions of protection from RNaseA1 by gp32 are marked by the triangles and protection from RNase T1 by the pentagonal symbols. The darker symbols denote stronger protection. Unmarked positions were not protected by either gp32 from phage source under the experimental conditions used.

As discussed above for Fig 3, computer-assisted and visual examinations of the RB69 *IC59-32 *nucleotide sequence predicted an RNA topology that was similar to the T4 gene *32 *translational operator, particularly with regards to presence of a putative RNA pseudoknot structure to the 5' side of the Shine-Dalgarno and UUAAA/UUAA sequence elements of the mRNA. We used 3 RNA modifying agents to test directly for intrinsic secondary or higher-order structure in the RB69-derived RNA: DMS, DEPC and RNase V1, respectively. Results are shown in Fig 5. We observed that the RB69-derived sequence from nucleotide position A(-1) through A(-45), relative to the initiator AUG, was hypersensitive to cleavage following DMS or DEPC treatment (Fig 5A) and relatively insensitive to cleavage by the dsRNA-specific RNase V1 (Fig 5B). These observations, which are summarized in Fig 6A, are consistent with the prediction that the A(-1) to A(-45) segment of the RB69 *IC59-32 *RNA region is intrinsically unstructured. In contrast, the segment of this RNA corresponding to the putative pseudoknot structure can accommodate a range of /RNA sequences. The interaction may also be subject to is hypersensitive to RNase V1 (Fig 5B) and less sensitive than the A(-1) to A(-45) segment to the 2 chemical agents used (Fig 5A). There was one unexpected observation in these experiments RB69 nucleotide position U(-20), which is located in the putatively unstructured portion of the RNA target (Fig 6A), appeared to be insensitive to DEPC modification (Fig 5A). Below, we show that another position in this segment, G(-10), is relatively insensitive to the ssRNA-specific RNase T1. Possibly, cleavage at U(-20) and G(-10) by RNA modifying agents is affected by RNA hairpin formation in the U(-8) to A(-21) sequence. The location of this putative hairpin, which is not predicted in the T4 RNA counterpart, is diagrammed in Fig 6A. In summary, the T4 gene 32 translational operator region and its putative counterpart from RB69 exhibit several topographical differences from each other, including an additional 6-nt sequence in RB69 that may contribute to RNA secondary structure formation in the RBS. Below, we show that the 2 regions also differ in their interactions with translational repressors.

### The footprints of T4 gp32 and RB69 gp32 on gene 32-specific RNA targets from T4 and RB69

**Figure 7:**
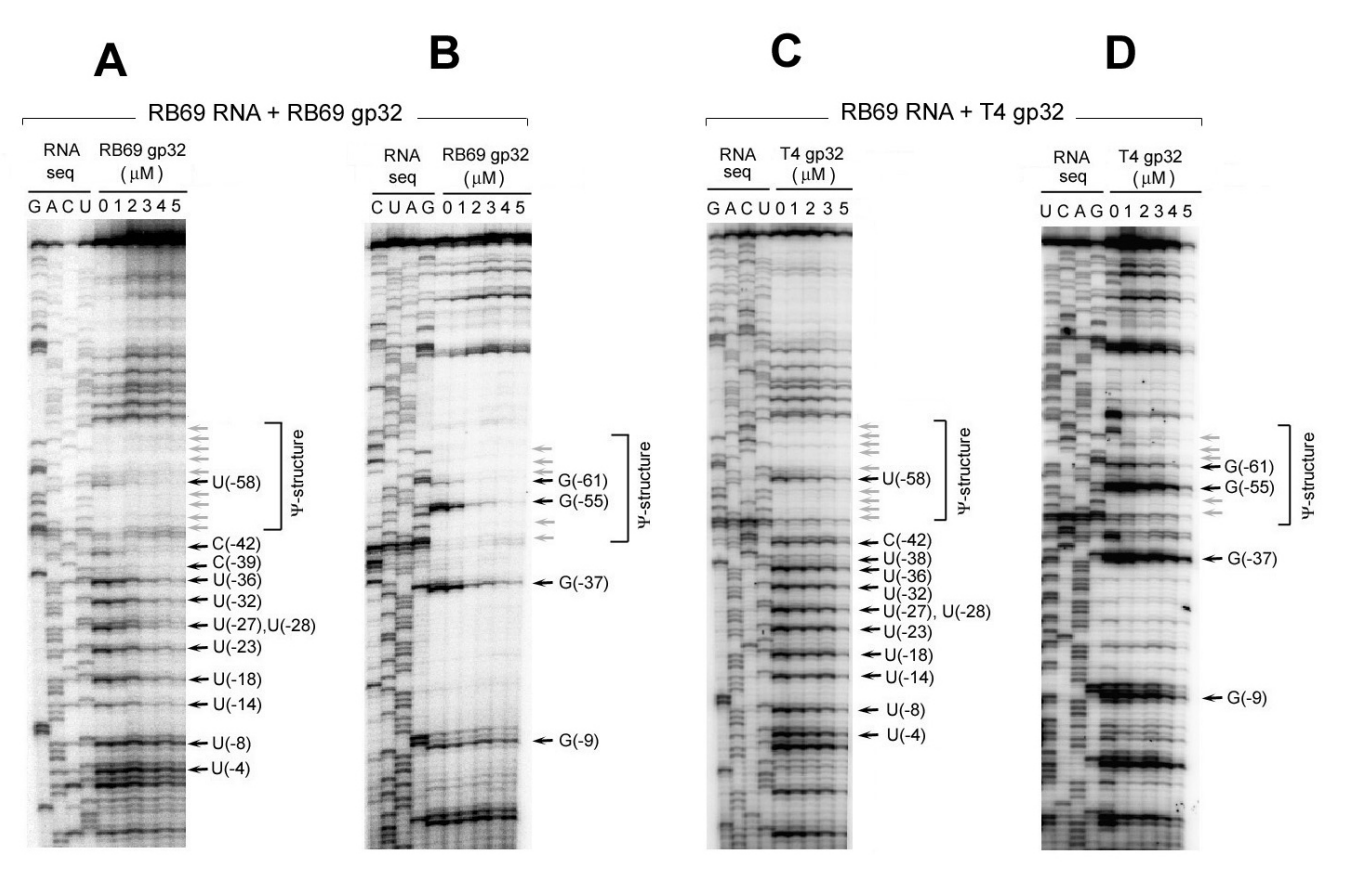
**In vitro footprinting of RB69 gene 32-specific RNA with purified RB69 gp32 (Panels A and B) and T4 gp32 (Panels C and D). **Preparation of RNA and proteins and experimental conditions for footprinting are described in METHODS. Horizontal arrows mark nucleotide positions (Fig 6B) that exhibited gp32-mediated protection from RNaseA (panels A and C) and RNase T1 (panels B and D). Darker arrows denote stronger protection. The results are summarized in Fig 6B.

**Figure 8:**
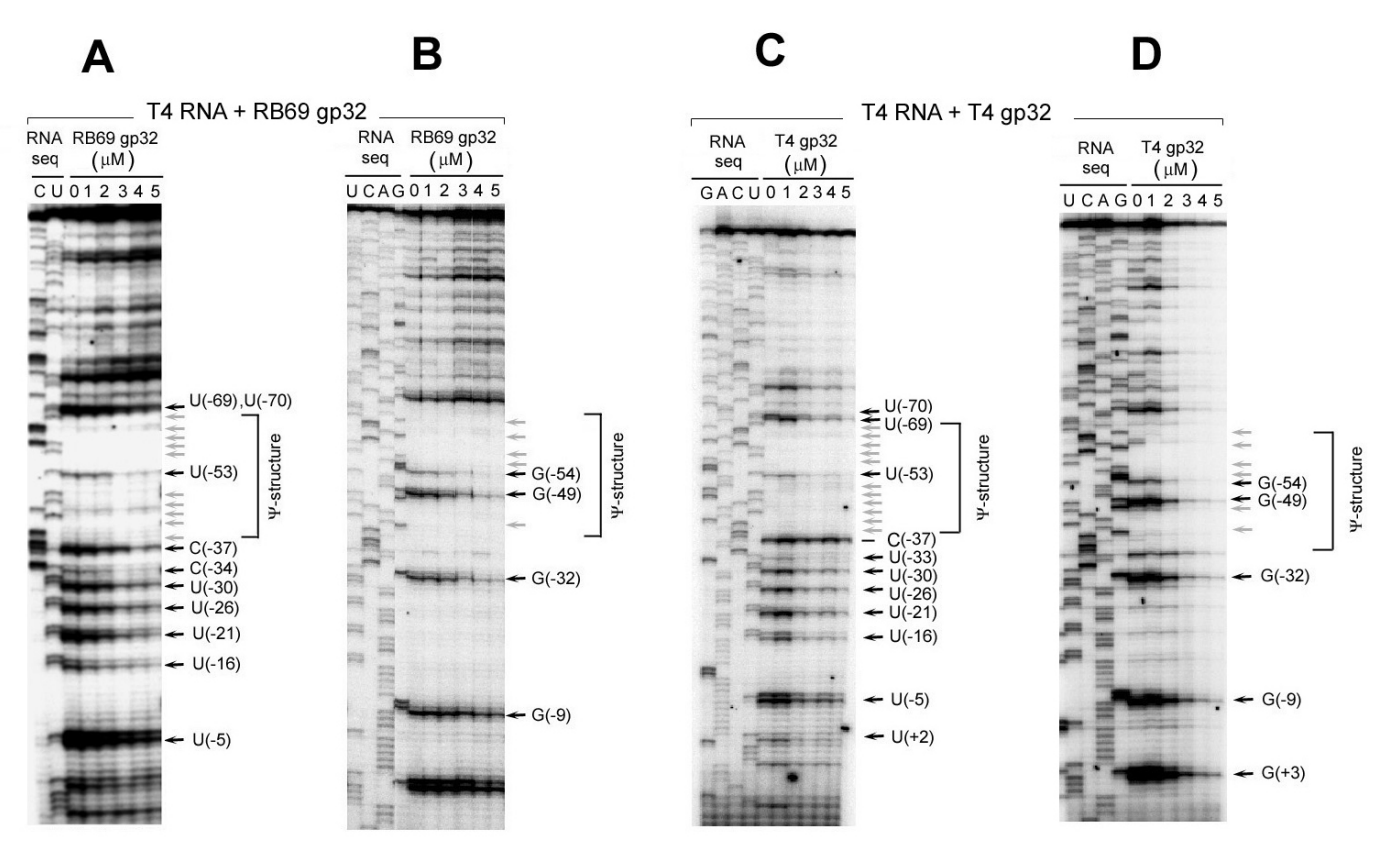
**In vitro footprinting of T4 gene32-specific RNA with purified RB69 gp32 (Panels A and C; RNase A) and T4 gp32 (Panels B and D; RNase T1). **Conditions for these experiments were identical to those described in Fig 7, except that the RNA substrate used for footprinting was derived from clones of T4 gene 32 rather than RB69 gene 32. See also Fig 6C for a summary.

We used the ssRNA-specific RNases A1 and T1 to determine the abilities of gp32 from the 2 phage systems to protect RNA targets from cleavage with these enzymes. These RNA footprinting studies also extended the information we obtained from treatments with DMS and DEPC about intrinsic structure of the RNA targets. Results are shown in Fig 7 for the RB69-derived RNA target and Fig 8 for the T4-derived target. Also, a summary of our observations from these experiments is presented on the RNA sequence charts in Fig 6B In the aggregate, our studies showed that T4 gp32 and RB69 gp32 contact RNA targets differently from each other, although the two proteins overlap in their RNA-binding properties. We highlight the following specific observations.

1. At the protein concentrations used (1–5 μM), the RB69 gp32 footprint on the RNA target from RB69 was 5 residues longer than the footprint of this protein on the T4-derived RNA target; however, the positions of the 2 footprints relative to the respective initiator AUG and 5' terminal boundary of the pseudoknot structure appeared to be identical (Fig 6)

2. As can be seen in Figs 7A and 7B, RB69 gp32 protected its own mRNA target strongly within the nucleotide segment between U(-14) and G(-61), and weakly in the segment from U(-2) to G(-9) In contrast, as seen in Figs 7C and 7D, T4 gp32 protected this RNA strongly only in the segment from C(-42) to G(-61)

3. As can be seen in Fig 8C and 8D, T4 gp32 protected the T4-derived RNA strongly in the G(+3) to U(-70) segment In contrast, RB69 gp32 protected this RNA target best in the U(-16) to U(-70) segment (Fig 7A and 7B)

It should be noted that the gp32 footprint sizes reported here are shorter than has been reported in studies that utilized higher concentrations of T4 gp32 with T4-specific RNA targets [16,32]. As stated earlier in this report (Fig 4), the higher gp32 concentrations (>5 μM) mask specificity differences between the T4 and RB69 proteins.

## Discussion

Phages T4 and RB69 are phylogenetically related to each other and encode homologous sets of DNA replication proteins that exhibit a significant degree of compatibility with each other's biological systems [22,24]. Despite such overlaps in function, we have commonly observed specificity differences between protein homologues from the 2 phage systems. For example, in plasmid-phage complementation assays, RB69 DNA polymerase (gp43) was observed to be just as effective as T4 gp43 in T4 DNA replication *in vivo*, whereas the T4 enzyme was less effective than its RB69 counterpart for RB69 DNA replication [22,33]. Also, the 2 DNA polymerases, like the 2 Ssb proteins compared here, are RNA-binding autogenous translational repressors that differ in RNA binding specificity and RNA target sequence. Studies with the T4 versions of gp43 and gp32 clearly show that the binding of these proteins to specific RNA is mutually exclusive with their binding to DNA [7,34]. So, conservation of the translational functions of these proteins may be related to conservation of their replication functions. Based on previous studies with RB69 gp43 [35], as well as the current study with RB69 gp32, we surmise that neither of these translational repressors possesses a domain that binds RNA exclusively. Rather, in both cases, the RNA binding site seems to be contained within the region of the protein that binds DNA. Thus, it is possible that in phage infected cells, specific RNA serves as a regulator of both the biosynthesis and replicative activities of these proteins.

In the purified system we have used to compare RNA footprints for gp32 from T4 and RB69 (Figs 6, 7, 8), we observed that the same RNA target could exhibit different patterns of protection depending on source of the Ssb protein. This observation suggests that the RNA-protein interaction is intrinsically flexible and can accommodate a range of RNA sequences as long as these sequences can be made to assume a certain configuration. In addition, the interaction could be subject to modulation by intra- and intermolecular protein-protein interactions of the repressor. In this regard, it is known that the extreme N-terminal segment (~20 residues) and C-terminal segment (~100 residues) of T4 gp32 have profound effects on the ssDNA binding activity, which is housed in the region bracketed by these 2 segments of the protein [19,36,37]. The N-terminal segment determines cooperative binding to ssDNA (through gp32-gp32 interactions) and the C-terminal segment has been implicated in interactions of gp32 with other phage induced proteins [38]. Possibly, the observed sequence divergence between the C-terminal domains of T4 gp32 and RB69 gp32 (Fig 2) was in part coupled to divergence of the mRNA targets during evolution of the 2 related translational repressors.

Although we cannot rule out the possibility that the C-terminal domain of gp32 influences specificity to RNA by interacting directly with this ligand, there are indications that this negatively charged segment of gp32 is a modulator of gp32 interactions with nucleic acids rather than a carrier of nucleic acid binding determinants. In particular, a small deletion that maps within this protein segment (Δ*32PR201*; Fig 2) exhibits altered specificity to other proteins but has no effects on autogenous control of gp32 synthesis *in vivo *[39]. Also, recent studies with purified RB69 gp32 implicated the C-terminal domain of this protein in regulating access of gp43 from the same phage to binding sites in the ssDNA-binding module of the Ssb protein [40]. It has also been shown that in T4, the ssDNA-binding module of gp32 forms specific crosslinks to gp59, the phage-induced primase-helicase loading protein [41,42]. Such observations suggest that the 2 nucleic-acid binding functions of gp32 may be subject to regulation by a combination of intra- and intermolecular protein-protein interactions involving the divergence-prone C-terminal domain. It would be particularly interesting to find out if the gp32 sequence divergence near the DNA binding residue Y106 (Fig 2) is important for RNA recognition. X-ray crystallographic studies [20] suggest that T4 gp32 residues T101-K110 constitute part of the ssDNA-binding surface of the protein, which includes Y84, Y99, Y106 and the nearby "LAST" motif (residues 110–114; 31). Also, as suggested by the 3D structure, these residues are located within or very close to the Zn-binding domain of the protein; ie, the putative "zinc-finger" sequence Cys77-X3-His-X5-Cys-X2-Cys90, which has counterparts in a number of RNA-binding proteins [40]. The construction and analysis of RB69-T4 gp32 chimeras could help to establish if the divergence near Y106 is responsible for the observed differences in RNA footprints between T4gp32 and RB69 gp32 (Figs 6, 7, 8).

In summary, we envisage that as a mediator of gp32's interactions with other phage induced proteins, the C-terminal domain of gp32 may co-diverge with its protein targets to maintain mutual recognition, and that structural plasticity of a conserved ssDNA-binding domain may allow an also diverging RNA target to establish rearranged contacts within a relatively conserved protein pocket. It is unclear if the 2 sets of divergence are interconnected, but together, they could facilitate the evolution of a high degree of diversity in how the synthesis and/or replication activity of this Ssb protein is regulated among phylogenetic relatives of T4. It will be important to find out if this diversity includes RNA ligands for gp32 that control the DNA-binding activity but not synthesis of gp32, or if autogenous translational repression has been replaced by other mechanisms for control of gene 32 in some T4 relatives. There is at least one reported example where evolution resulted in lack of RNA binding function in the Ssb protein of an M13-like phage [43]. Also, a scan of available genomic sequences for T4-like phages  reveals a high degree of sequence divergence in the putative translational operator regions of the corresponding gene 32 regions. In one case, phage RB49 (GenBank NC_005066), it has been reported that there are no indications that an RNA pseudoknot structure exists in the putative TIR for gene 32, although the UUAA/UUAAA sequence units are conserved in the RB49 *IC59-32 *region [44]. It remains to be seen if gp32 from this and other T4 like phages that appear to lack the RNA pseudoknot do bind their respective TIR regions or repress their own translation.

Finally, we should comment about *ORF32.1 *(Fig 1) and its possible relevance to evolution of the mRNA target for gp32. This ORF is present in some T4-like genomes (eg T4 and GenBank Ac No AF033323) and absent in others (eg, RB69 and GenBank Ac No AY310907). Recently, it was shown that T4 *ORF 32.1 *encodes a Seg-type (G1Y-YIG family) homing endonuclease (now named SegG) that mediates its own transfer, along with T4 gene 32, to the *ORF32.1*-less genome of phage T2 in T4 × T2 genetic crosses. We note that the 5' terminal sequence of the RNA pseudoknot for T4 gp32 translational control overlaps the reading frame of the segG gene, in addition to being very similar (~83% identity) to the corresponding segment of the pseudoknot sequence of RB69, which lacks a segG gene (Fig 3). Possibly, this portion of the RNA pseudoknot preexisted the entry of an *ORF32.1*-like sequence element into the gene *59-32 *intercistronic region of a T4 progenitor and that the modern day *segG *gene (*ORF32.1*) may be a chimera consisting of an extension of the parental *segG *reading frame into the recipient genome's pseudoknot sequence. Such lateral transfer events and subsequent mutation may have profound influences on evolution of the RNA binding functions of proteins that have relaxed sequence but stringent structural requirements for their RNA target.

## Competing interests

None declared.

## Authors' contributions

Jamilah Borjac-Natour: Conducted most of the experimental work and initial data analysis and prepared summaries; wrote the first draft and participated in subsequent revisions of the manuscript. Vasiliy Petrov: Conducted independent analysis of data and generated summaries and composite figures for presentation in the manuscript. Participated in revision of the manuscript during later stages of preparation. Jim Karam: Directed the study, evaluated results on an ongoing basis, worked closely with the coauthors during preparation of Figures, played a major role during revision of manuscript drafts and communicated the manuscript to the journal.

## References

[B1] Alberts BM, Frey L (1970). T4 bacteriophage gene 32: a structural protein in the replication and recombination of DNA. Nature.

[B2] Kreuzer KN, Morrical SW (1994). in Initiation of DNA Replication. Molecular Biology of Bacteriophage T4.

[B3] Mosig G (1994). in Recombination Models and Pathways. Molecular Biology of Bacteriophage T4.

[B4] Bleuit JS, Xu H, Ma Y, Wang T, Liu J, Morrical SW (2001). Mediator proteins orchestrate enzyme-ssDNA assembly during T4 recombination-dependent DNA replication and repair. Proc Natl Acad Sci USA.

[B5] Lefebvre SD, Wong ML, Morrical SW (1999). Simultaneous interactions of bacteriophage T4 DNA replication proteins gp59 and gp32 with single-stranded (ss) DNA. Co-modulation of ssDNA binding activities in a DNA helicase assembly intermediate. J Biol Chem.

[B6] Beernink HT, Morrical SW (1999). RMPs: recombination/replication mediator proteins. Trends Biochem Sci.

[B7] Miller ES, Karam JD, Spicer E (1994). in Control of Translation Initiation: mRNA Structure and Protein Repressors. Molecular Biology of Bacteriophage T4.

[B8] Model P, McGill C, Mazur B, Fulford WD (1982). The replication of bacteriophage f1: gene V protein regulates the synthesis of gene II protein. Cell.

[B9] Fulford W, Russel M, Model P (1986). Aspects of the growth and regulation of the filamentous phages. Prog Nucleic Acid Res Mol Biol.

[B10] von Hippel PH, Kowalczykowski SC, Lonberg N, Newport JW, Paul LS, Stormo GD, Gold L (1982). Autoregulation of gene expression. Quantitative evaluation of the expression and function of the bacteriophage T4 gene 32 (single-stranded DNA binding) protein system. J Mol Biol.

[B11] Kowalczykowski SC, Lonberg N, Newport JW, Paul LS, von Hippel PH (1980). On the thermodynamics and kinetics of the cooperative binding of bacteriophage T4-coded gene 32 (helix destabilizing) protein to nucleic acid lattices. Biophys J.

[B12] Newport JW, Lonberg N, Kowalczykowski SC, von Hippel PH (1981). Interactions of bacteriophage T4-coded gene 32 protein with nucleic acids. II. Specificity of binding to DNA and RNA. J Mol Biol.

[B13] Lemaire G, Gold L, Yarus M (1978). Autogenous translational repression of bacteriophage T4 gene 32 expression in vitro. J Mol Biol.

[B14] Russel M, Gold L, Morrissett H, O'Farrell PZ (1976). Translational, autogenous regulation of gene 32 expression during bacteriophage T4 infection. J Biol Chem.

[B15] Shamoo Y, Tam A, Konigsberg WH, Williams KR (1993). Translational repression by the bacteriophage T4 gene 32 protein involves specific recognition of an RNA pseudoknot structure. J Mol Biol.

[B16] McPheeters DS, Stormo GD, Gold L (1988). Autogenous regulatory site on the bacteriophage T4 gene 32 messenger RNA. J Mol Biol.

[B17] Krisch HM, Allet B (1982). Nucleotide sequences involved in bacteriophage T4 gene 32 translational self-regulation. Proc Natl Acad Sci USA.

[B18] Fulford W, Model P (1984). Specificity of translational regulation by two DNA-binding proteins. J Mol Biol.

[B19] Williams K, Shamoo Y, Spicer EK, Coleman J, Konigsberg W (1994). in Correlation Structure to Function in Proteins: T4 Gp32 as a prototype. Molecular Biology of Bacteriophage T4.

[B20] Shamoo Y, Friedman AM, Parsons MR, Konigsberg WH, Steitz TA (1995). Crystal structure of a replication fork single-stranded DNA binding protein (T4 gp32) complexed to DNA. Nature.

[B21] Studier FW, Moffatt BA (1986). Use of bacteriophage T7 RNA polymerase to direct selective high-level expression of cloned genes. J Mol Biol.

[B22] Wang CC, Yeh LS, Karam JD (1995). Modular organization of T4 DNA polymerase. Evidence from phylogenetics. J Biol Chem.

[B23] Hsu T, Karam JD (1990). Transcriptional mapping of a DNA replication gene cluster in bacteriophage T4. Sites for initiation, termination, and mRNA processing. J Biol Chem.

[B24] Yeh LS, Hsu T, Karam JD (1998). Divergence of a DNA replication gene cluster in the T4-related bacteriophage RB69. J Bacteriol.

[B25] Wang CC, Pavlov A, Karam JD (1997). Evolution of RNA-binding specificity in T4 DNA polymerase. J Biol Chem.

[B26] Bittner M, Burke RL, Alberts BM (1979). Purification of the T4 gene 32 protein free from detectable deoxyribonuclease activities. J Biol Chem.

[B27] Liang YM, Wei RX, Hsu T, Alford C, Dawson M, Karam J (1988). Autogenous regulation of the regA gene of bacteriophage T4: derepression of translation. Genetics.

[B28] Wang CC (1997). Structural organization and RNA-binding properties of the DNA polymerases of bacteriophages T4 and RB69. Dissertatin in partial fulfillment of requirements for Ph.D. Department of Biochemistry. Tulane University Health Sciences Center.

[B29] Petrov VM, Karam JD (2002). RNA determinants of translational operator recognition by the DNA polymerases of bacteriophages T4 and RB69. Nucleic Acids Res.

[B30] Schagger H, von Jagow G (1987). Tricine-sodium dodecyl sulfate-polyacrylamide gel electrophoresis for the separation of proteins in the range from 1 to 100 kDa. Anal Biochem.

[B31] Waidner LA, Flynn EK, Wu M, Li X, Karpel RL (2001). Domain effects on the DNA-interactive properties of bacteriophage T4 gene 32 protein. J Biol Chem.

[B32] McPheeters DS, Gosch G, Gold L (1988). Nucleotide sequences of the bacteriophage T2 and T6 gene 32 mRNAs. Nucleic Acids Res.

[B33] Dressman HK, Wang CC, Karam JD, Drake JW (1997). Retention of replication fidelity by a DNA polymerase functioning in a distantly related environment. Proc Natl Acad Sci USA.

[B34] Pavlov AR, Karam JD (1994). Binding specificity of T4 DNA polymerase to RNA. J Biol Chem.

[B35] Petrov VM, Ng SS, Karam JD (2002). Protein determinants of RNA binding by DNA polymerase of the T4-related bacteriophage RB69. J Biol Chem.

[B36] Villemain JL, Ma Y, Giedroc DP, Morrical SW (2000). Mutations in the N-terminal cooperativity domain of gene 32 protein alter properties of the T4 DNA replication and recombination systems. J Biol Chem.

[B37] Shamoo Y, Williams KP, Konigsberg W (1994). in The function of Zinc(II) in Gene 32 Protein (Gp32). Molecular Biology of Bacteriophage T4.

[B38] Hurley JM, Chervitz SA, Jarvis TC, Singer BS, Gold L (1993). Assembly of the bacteriophage T4 replication machine requires the acidic carboxy terminus of gene 32 protein. J Mol Biol.

[B39] Karam JD, Leach M, Heere LJ (1979). Functional interactions beween the DNA ligase of Escherichia coli and components of the DNA metabolic apparatus of T4 bacteriophage. Genetics.

[B40] Shamoo Y, Webster KR, Williams KR, Konigsberg WH (1991). A retrovirus-like zinc domain is essential for translational repression of bacteriophage T4 gene 32. J Biol Chem.

[B41] Ishmael FT, Alley SC, Benkovic SJ (2001). Identification and mapping of protein-protein interactions between gp32 and gp59 by cross-linking. J Biol Chem.

[B42] Ishmael FT, Trakselis MA, Benkovic SJ (2003). Protein-protein interactions in the bacteriophage T4 replisome. The leading strand holoenzyme is physically linked to the lagging strand holoenzyme and the primosome. J Biol Chem.

[B43] Zaman GJ, Kaan AM, Schoenmakers JG, Konings RN (1992). Gene V protein-mediated translational regulation of the synthesis of gene II protein of the filamentous bacteriophage M13: a dispensable function of the filamentous-phage genome. J Bacteriol.

[B44] Desplats C, Dez C, Tetart F, Eleaume H, Krisch HM (2002). Snapshot of the genome of the pseudo-T-even bacteriophage RB49. J Bacteriol.

[B45] Liu Q, Belle A, Shub DA, Belfort M, Edgell DR (2003). SegG endonuclease promotes marker exclusion and mediates co-conversion from a distant cleavage site. J Mol Biol.

[B46] Shamoo Y, Abdul-Manan N, Patten AM, Crawford JK, Pellegrini MC, Williams KR (1994). Both RNA-binding domains in heterogenous nuclear ribonucleoprotein A1 contribute toward single-stranded-RNA binding. Biochemistry.

